# Effect of (−)-Epigallocatechin Gallate on Activation of JAK/STAT Signaling Pathway by Staphylococcal Enterotoxin A

**DOI:** 10.3390/toxins13090609

**Published:** 2021-08-29

**Authors:** Yuko Shimamura, Rina Noaki, Ami Kurokawa, Mio Utsumi, Chikako Hirai, Toshiyuki Kan, Shuichi Masuda

**Affiliations:** 1 School of Food and Nutritional Sciences, University of Shizuoka, 52-1 Yada, Suruga-ku, Shizuoka 422-8526, Japan; shimamura@u-shizuoka-ken.ac.jp (Y.S.); s19205@u-shizuoka-ken.ac.jp (R.N.); s17205@u-shizuoka-ken.ac.jp (A.K.); s16204@u-shizuoka-ken.ac.jp (M.U.); gp1848@u-shizuoka-ken.ac.jp (C.H.); 2 Department of Synthetic Organic & Medicinal Chemistry, School of Pharmaceutical Sciences, University of Shizuoka, 52-1 Yada, Suruga-ku, Shizuoka 422-8526, Japan; kant@u-shizuoka-ken.ac.jp

**Keywords:** staphylococcal enterotoxin A, gp130, STAT3, (−)-epigallocatechin gallate

## Abstract

Staphylococcal enterotoxin A (SEA), which is a superantigen toxin protein, binds to cytokine receptor gp130. Gp130 activates intracellular signaling pathways, including the Janus kinase/signal transducers and activators of transcription (JAK/STAT) pathway. The effects of SEA on the JAK/STAT signaling pathway in mouse spleen cells were examined. After treatment with SEA, mRNA expression levels of interferon gamma (IFN-γ) and suppressor of cytokine-signaling 1 (SOCS1) increased. SEA-induced IFN-γ and SOCS1 expression were decreased by treatment with (−)-epigallocatechin gallate (EGCG). The phosphorylated STAT3, Tyr705, increased significantly in a SEA concentration-dependent manner in mouse spleen cells. Although (−)-3″-Me-EGCG did not inhibit SEA-induced phosphorylated STAT3, EGCG and (−)-4″-Me-EGCG significantly inhibited SEA-induced phosphorylated STAT3. It was thought that the hydroxyl group at position 3 of the galloyl group in the EGCG was responsible for binding to SEA and suppressing SEA-induced phosphorylation of STAT3. Through protein thermal shift assay in vitro, the binding of the gp130 receptor to SEA and the phosphorylation of STAT3 were inhibited by the interaction between EGCG and SEA. As far as we know, this is the first report to document that EGCG inhibits the binding of the gp130 receptor to SEA and the associated phosphorylation of STAT3.

## 1. Introduction

*Staphylococcus aureus* is a gram-positive coccus that is present in the human skin and nasal cavity. Staphylococcal enterotoxin A (SEA) frequently causes staphylococcal food poisoning. In addition, SEA is a superantigen toxin protein that can lead to the potentially fatal production of inflammatory cytokines [1]. As a superantigen, SEA is crosslinked with major histocompatibility complex (MHC) Class II molecules and T cell receptors, inducing the massive activation of T cells and consequently producing large amounts of cytokines that cause various diseases [2]. SEA binds to the cytokine receptor gp130 [3], which is known to activate the Janus activated kinase (JAK) and signal transducer and activator of transcription (STAT) signaling pathway [4]. The mechanism of activation of the JAK/STAT pathway by the binding of SEA to the glycoprotein gp130 has not yet been clarified.

In adipocytes and hepatocytes, interleukin 6 (IL-6) binds to gp130 and induces suppressor of cytokine-signaling 1 (SOCS1) expression via the JAK/STAT pathway. This inhibits tyrosine phosphorylation, thereby inhibiting the action of insulin [5,6]. SOCS1 regulates both the adaptive and the innate immune responses [7]. *S. aureus* infection enhances SOCS1 expression in mice [8] and activates JAK/STAT signaling, including STAT3, in murine macrophage-like RAW264.7 cells [9]. Many inflammation-related diseases, such as Crohn’s disease, pleurisy, and psoriasis, are caused by overactivation of the JAK/STAT pathway [10]. Controlling overexpression and activation of the JAK/STAT pathway represents a new strategy for treating these chronic inflammatory responses.

**Figure 1:**
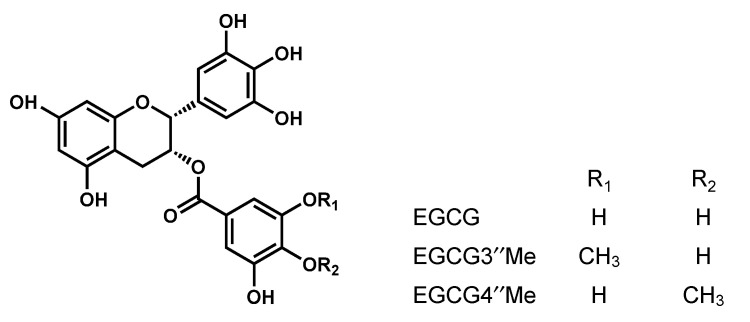
Structure of (−)-epigallocatechin gallate (EGCG) and methylated EGCG.

It is known that polyphenols exert various physiological functions because of the diversity of their structures [11]. Catechins, which are the major functional ingredients of green tea (*Camellia sinensis*), have been reported to have various actions, including antibacterial action, anticancer action, and antioxidant action [12]. The main catechins are (−)-epicatechin, (−)-epicatechin-gallate, (−)-epigallocatechin, and (−)-epigallocatechin gallate (EGCG). EGCG undergoes substantial in vivo changes into species containing methylated compounds. Recent studies have shown that methylated EGCG has many biological activities. Methylated EGCG [(−)-3”-Me-EGCG and (−)-4”-Me-EGCG] (Figure 1) inhibit Type I and Type IV allergies more effectively than EGCG [13,14]. Dietary catechins have been widely reported to suppress inflammatory response, and are becoming potential therapeutic agents for inflammatory diseases. Catechins activate/inhibit several signaling pathways, mainly by direct interaction with specific protein targets [15]. We previously reported that EGCG binds to SEA [16] and inhibits toxic activity via direct interaction with SEA [17]. Therefore, EGCG may have the ability to inhibit binding of SEA to the gp130 receptor. However, changes in STAT phosphorylation associated with activation of the gp130 receptor due to the binding of SEA to EGCG have not been clarified. This study aimed to clarify the mechanism of SEA-induced JAK/STAT pathway activation via the binding of SEA and gp130. We also examined the inhibitory effects of EGCG on SEA-induced STAT activation and cytokine production, using mouse spleen cells.

## 2. Results

### 2.1. SEA-Induced JAK/STAT Signaling Pathway Gene Expression

**Figure 2:**
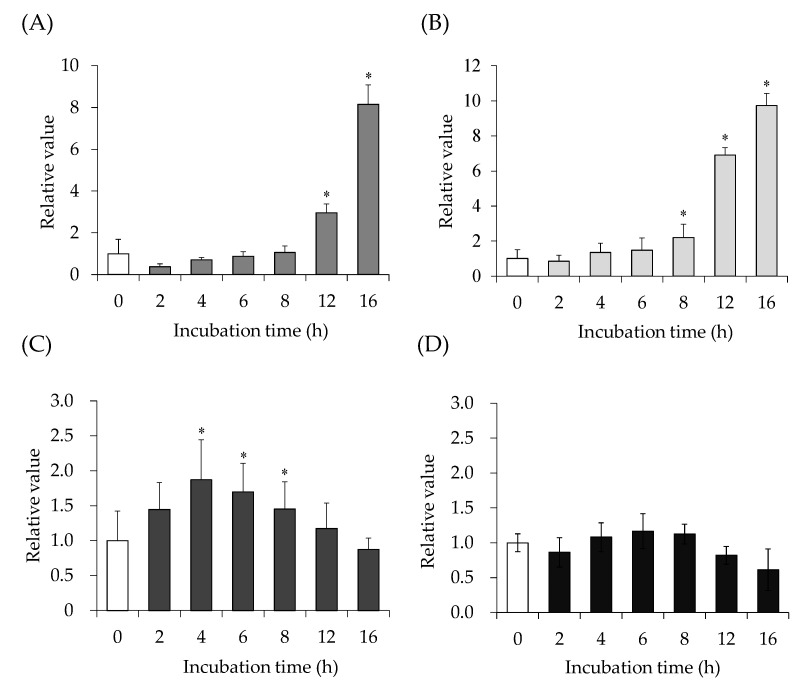
Time-dependent changes in (**A**) interferon gamma (IFN-γ), (**B**) suppressor of cytokine-signaling 1 (SOCS1), (**C**) signal transducers and activators of transcription 3 (STAT3), and (**D**) interleukin 6 (IL-6) mRNA expression induced by staphylococcal enterotoxin A (SEA) in mouse spleen cells. mRNA expression induced by SEA (50 ng/mL) was detected using real-time reverse transcription polymerase chain reaction (RT-PCR). Expression was normalized to the hypoxanthine-guanine phosphoribosyl transferase gene. Fold change was determined relative to the control at incubation time zero. The values represent the mean ± standard deviation (SD) of the four independent experiments. *, The significance level was *p* < 0.05 compared with the control.

The effects of SEA on the JAK/STAT signaling pathway in mouse spleen cells were examined. The time course of interferon gamma (IFN-γ), SOCS1, STAT3, and IL-6 mRNA expression levels in mouse spleen cells after SEA treatment was measured using reverse transcription polymerase chain reaction (RT-PCR). After treatment with SEA (50 ng/mL), mRNA expression levels of IFN-γ (Figure 2A) and SOCS1 (Figure 2B) increased significantly at 8–16 h. STAT3 expression increased significantly 4–8 h after SEA exposure and then decreased (Figure 2C). The expression level of IL-6 did not change with or without SEA (Figure 2D).

### 2.2. Inhibitory Effect of Catechin on SEA-Induced JAK/STAT Signaling Pathway Gene Expression

**Figure 3:**
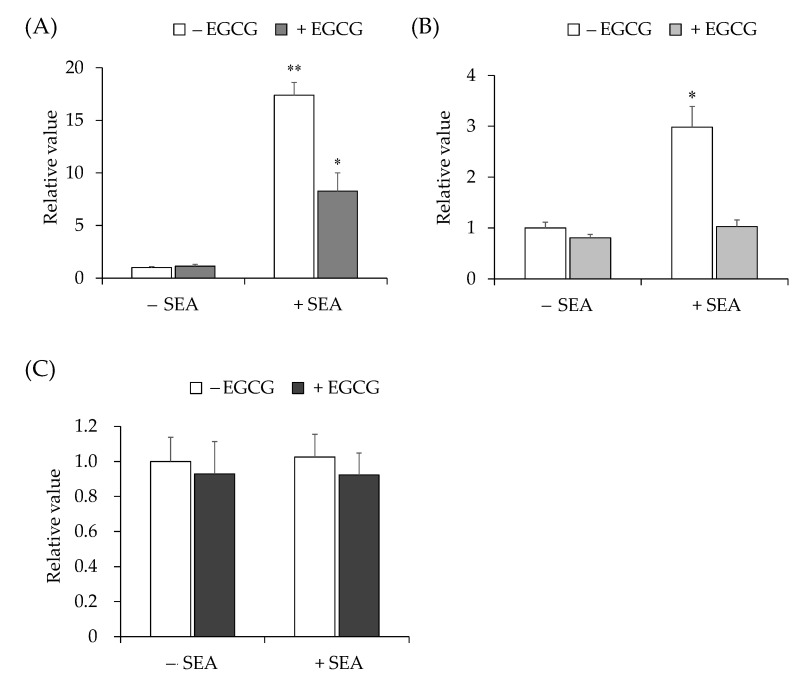
Effects of EGCG on SEA-induced mRNA expression of (**A**) IFN-γ, (**B**) SOCS1, and (**C**) STAT3 in mouse spleen cells. With (+SEA) or without (−SEA) SEA (50 ng/mL) and EGCG (0.05 mM) for 16 h, induced mRNA expression levels were detected using real-time RT-PCR. Expression was normalized to the hypoxanthine–guanine phosphoribosyl transferase gene. Fold change was determined relative to the control without SEA. The values represent the mean ± SD of the three independent experiments. *, *p* < 0.05 vs. control without SEA. **, *p* < 0.01 vs. control without SEA.

The effects of EGCG on the SEA-induced JAK/STAT signaling pathway were examined. Mouse spleen cells were pre-incubated with SEA (50 ng/mL) and EGCG (0.05 mM) at 37 °C for 2 h, and incubated at 37 °C, 5% CO_2_ for 16 h. SEA-induced IFN-γ (Figure 3A) and SOCS1 (Figure 3B) expression levels were decreased by EGCG. The expression level of STAT3 did not change with or without SEA or EGCG (Figure 3C).

### 2.3. Effect of SEA on Phosphorylation of STAT3

**Figure 4:**
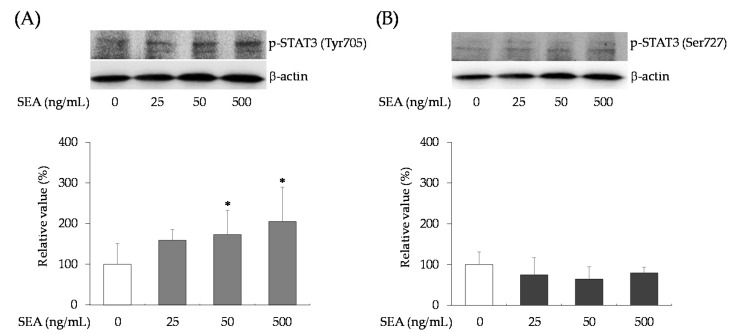
Effects of SEA on (**A**) phosphorylated (p)-STAT3 tyrosine (Tyr705), and (**B**) p-STAT3 serine (Ser727). Mouse spleen cells were treated with SEA (25, 50, and 500 ng/mL) for 6 h. Total cell lysates were prepared and subjected to Western blot analyses with antibodies against p-STAT3 Tyr705 or p-STAT3 Ser727. Protein levels of p-STAT3 Tyr705 or p-STAT3 Ser727were expressed as relative to a background corrected protein band normalized to β-actin. The values represent the mean ± SD of the three independent experiments. *, *p* < 0.05, compared against control without SEA.

The expression of phosphorylated STAT3 (Tyr705/Ser727) in mouse spleen cells treated with SEA (500 ng/mL) for 6 h was examined using Western blot analysis. Phosphorylated STAT3 Tyr705 increased significantly in a SEA concentration-dependent manner (Figure 4A), but phosphorylated STAT3 Ser727 showed no change with SEA treatment (Figure 4B).

### 2.4. Effects of Catechin on SEA-Induced Phosphorylated STAT3

**Figure 5:**
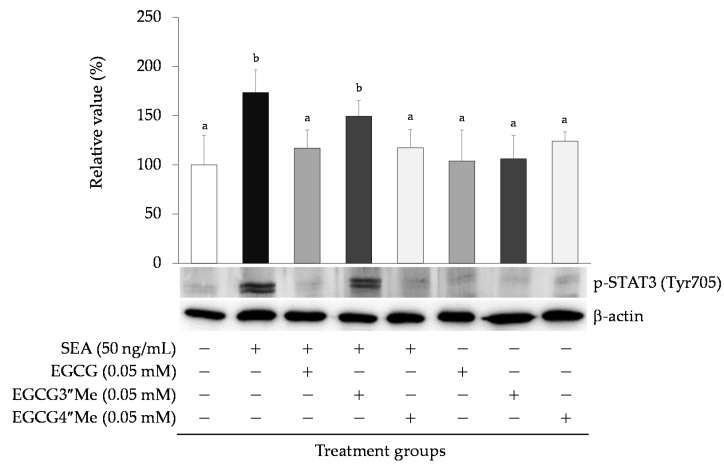
Effects of EGCG and methylated EGCG [(−)-3″-Me-EGCG and (−)-4″-Me-EGCG] on SEA-induced phosphorylated p-STAT3 Tyr705. Mouse spleen cells were treated with SEA (50 ng/mL) and EGCG or methylated EGCG (0.05 mM) for 6 h. Total cell lysate was applied to sodium dodecyl sulfate polyacrylamide gel electrophoresis (SDS-PAGE) and visualized using Western blot analysis with anti-p-STAT3 Tyr705 and β-actin antibodies. There was a significant difference between different letters (Tukey–Kramer test, *p* < 0.05). The values represent the mean ± SD of the three independent experiments.

The effects of EGCG (0.05 mM) on SEA-induced phosphorylated STAT3 Tyr705 in mouse spleen cells were examined. EGCG significantly inhibited SEA-induced phosphorylated STAT3 Tyr705 (Figure 5). To investigate the role of the galloyl group on SEA-induced phosphorylated STAT3 in more detail, the inhibitory effect of the hydroxyl group of the galloyl group of EGCG on SEA-induced phosphorylated STAT3 was examined using methylated catechins. Although (−)-3″-Me-EGCG did not inhibit SEA-induced phosphorylated STAT3, EGCG and (−)-4″-Me-EGCG significantly inhibited SEA-induced phosphorylated STAT3 (Figure 5).

### 2.5. Interaction between SEA and Catechin

**Figure 6:**
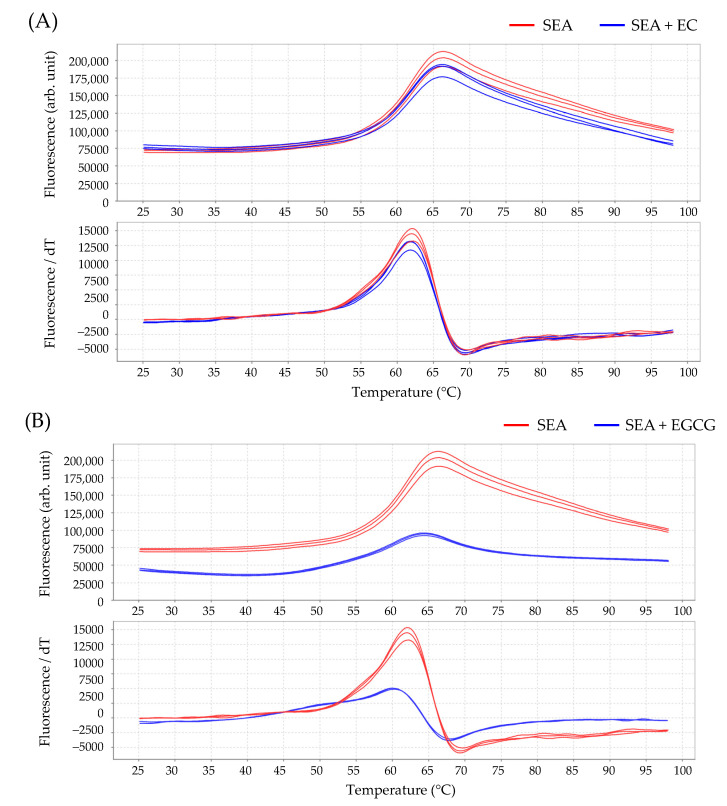
Melting profile of staphylococcal enterotoxin A. Melting profile of SEA with (**A**) (−)-epicatechin (EC), and (**B**) EGCG. The upper figure shows the thermal denaturation profile of proteins. The lower figure shows the profile of the derivative of fluorescence emission as a function of temperature (dF/dT). SEA (5.0 μg/mL) and ligand (250 μM each for EC and EGCG) were incubated at 37 °C for 2 h before using thermal shift assay.

Interactions between SEA and catechins (EC or EGCG) were analyzed using protein thermal shift assay. There was no significant difference between the melting temperature (*T*_m_) value of SEA and SEA treated with EC (61.86 ± 0.05 °C) (Table 1, Figure 6A). On the other hand, the *T*_m_ value of SEA treated with EGCG (60.13 ± 0.09 °C) shifted to a lower temperature, compared with SEA treatment only (62.08 ± 0.07 °C) (Table 1, Figure 6B). This indicates that SEA and EGCG interacted; this interaction could be confirmed using protein thermal shift assay.

### 2.6. Interaction between SEA/EGCG and Gp130

**Figure 7:**
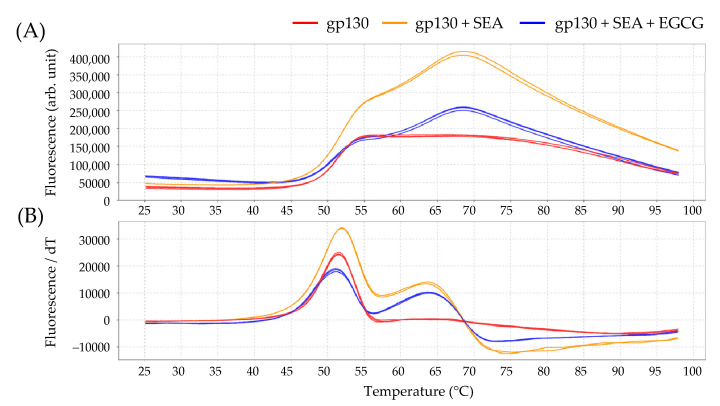
Melting profile of gp130 in the presence of SEA and EGCG with (**A**) the thermal denaturation profile of proteins, and (**B**) the profile of the fluorescence derivative as a function of temperature (dF/dT). SEA (5.0 μg/mL) and ligand (250 μM EGCG and 1.0 μM gp130) were incubated at 37 °C for 2 h before using thermal shift assay.

The interaction between EGCG-reacted SEA and gp130 was analyzed using protein thermal shift assay. The *T*_m_ value of gp130 with SEA (51.96 ± 0.01 °C) shifted to a higher temperature than the *T*_m_ of gp130 with EGCG-reacted SEA (51.09 ± 0.02 °C) (Table 2, Figure 7). The binding of the gp130 receptor to SEA was inhibited by the interaction between EGCG and SEA.

## 3. Discussion

This study investigated the mechanism of SEA-induced JAK/STAT signaling pathway activation. It was previously reported that SEA binds to the gp130 receptor using the C-terminal domain [3]. The gp130 receptor complex activates the JAK/STAT signaling pathway, leading to phosphorylation and activation of the transcription factor STAT3 [18]. Therefore, JAK/STAT pathway-related genes, induced by SEA in mouse spleen cells, were examined using real-time RT-PCR. SEA was used at a concentration that was not cytotoxic [19]. SEA exposure increased mRNA expression levels of IFN-γ (Figure 2A), SOCS1 (Figure 2B), and STAT3 (Figure 2C). STAT3 expression increased significantly 4–8 h after SEA exposure and then decreased (Figure 2C). The expression level of IL-6 did not change with or without SEA (Figure 2D). Since gp130 is expressed in most cell types in the body, SEA may target and regulate gp130 in cells that do not express MHC Class II or T cell antigen receptors. It may further regulate the local inflammation and immune response caused by *S. aureus* infection, for example, food poisoning, chronic skin infection, pneumonia, and infective endocarditis. SEA activates the same signaling receptors as IL-6 (gp130) [20]. Thus, SEA is expected to have similar effects to IL-6. SEA induced INF-γ expression but not IL-6 expression, suggesting that the SEA-induced JAK/STAT3 signaling pathway is not IL-6-dependent.

The development of Type II diabetes in humans is associated with excessive weight and obesity and is caused by overexpression of the SOCS protein [6]. Type II diabetics carry a high risk of *S. aureus* colonization and overt infections. Chronic stimulation of rabbits with *S. aureus* superantigen toxic shock syndrome toxin-1 has been reported to cause impaired glucose tolerance throughout the body [21]. It has been suggested that *S. aureus* superantigen SEA might induce Type II diabetes by increasing the expression level of SOCS1.

We previously reported that EGCG binds to SEA [16]. Thus, it was thought that EGCG may have the ability to inhibit gp130 binding to SEA. However, changes in SEA-induced JAK/STAT pathway-related gene expression due to the binding of SEA to EGCG have not been clarified. Therefore, the effects of EGCG on the expression of SEA-induced JAK/STAT pathway-related gene expression in mouse spleen cells were examined. Expression of SEA-induced IFN-γ (Figure 3A) and SOCS1 (Figure 3B) was decreased by EGCG. Some polyphenols have been reported to inhibit IL-6-induced JAK/STAT3 signaling pathway [22]. Some natural agents with chemopreventive effects are very effective in suppressing the activation of STAT3 [23]. Curcumin has been shown to inhibit the activity of proteins involved in STAT3 phosphorylation, such as IL-6 [24,25]. Resveratrol was found to inhibit IL-6-induced activation of STAT3 in human multiple myeloma cells and endothelial cells [26,27]. However, STAT3 expression level did not change with or without SEA or EGCG at 16 h (Figure 3C). When EGCG was added at 4 and 8 h, EGCG slightly decreased the expression level of SEA-induced STAT3 but no significant difference was observed (data not shown). SEA was believed to induce STAT3 phosphorylation without affecting STAT3 gene expression.

STAT3 is a potential transcription factor present in the cytoplasm. When activated by tyrosine phosphorylation, STAT3 dimers translocate to the nucleus, bind to nuclear DNA, and regulate the transcription of target genes. Phosphorylation of STAT3 is primarily mediated by activation of the JAK non-receptor protein tyrosine kinase family [28,29]. The major phosphorylation sites of STAT3 are tyrosine (Tyr705) and serine (Ser727) residues. Therefore, we examined the expression of phosphorylated STAT3 (Tyr705/Ser727) in mouse spleen cells treated with SEA. SEA did not induce STAT3 gene expression, but significantly upregulated STAT3 Tyr705 phosphorylation (Figure 4A). SEA did not induce phosphorylation of STAT3 Ser727 (Figure 4B). Although one of the essential events of activation of STAT3 is phosphorylation of its Tyr705 residue, some studies have reported that STAT3 Ser727 phosphorylation negatively modulated Tyr705 phosphorylation [30,31]. In addition, serine phosphorylation has been shown to have no effect on either STAT translocation [32] or DNA binding [33]. Regardless of the phosphorylation status of the Ser727 residue, activation of the Tyr705 residue by SEA alone appeared to be sufficient for nuclear translocation and DNA binding activity.

The effect of EGCG on SEA-induced phosphorylated STAT3 Tyr705 was examined. EGCG significantly inhibited the production of SEA-induced phosphorylated STAT3. To investigate the role of the galloyl group of SEA-induced phosphorylated STAT3 in more detail, the effect of methylated EGCG on SEA-induced phosphorylated STAT3 Tyr705 was examined. Although (−)-3″-Me-EGCG did not inhibit the production of SEA-induced phosphorylated STAT3, (−)-4″-Me-EGCG significantly inhibited the production of SEA-induced phosphorylated STAT3. Our previous studies have shown that EGCG and (−)-4″-Me-EGCG interact with SEA, and (−)-3″-Me-EGCG does not interact with SEA [16]. The difference between EGCG and (-) -3″-Me-EGCG is the presence of a methyl group at the 3″ position of the galloyl group (Figure 1). This information suggests that the hydroxyl group at the 3-position of the galloyl group in the catechin was responsible for its binding affinity with SEA. It was thought that the hydroxyl group at the 3-position of the galloyl group in EGCG was responsible for binding to SEA and suppressing the SEA-induced phosphorylation of STAT3.

In order to clarify that this activation is caused by interaction between SEA and gp130, interactions between SEA and the catechins EGCG and EC were analyzed using protein thermal shift assay. Although EGCG and SEA interacted, EC and SEA did not interact. These results support those of our previous study, which indicate that SEA binds to a galloyl group [16]. We further analyzed the interaction between EGCG-reacted SEA and the gp130 receptor activating STAT3 using protein thermal shift assay. The binding of the gp130 receptor to SEA and the phosphorylation of STAT3 were inhibited by the interaction between EGCG and SEA.

STAT3 is an oncogene that promotes cell survival, proliferation, and the progression of cancer cells. EGCG is the major catechin in green tea; it is recognized as an important chemopreventive agent and modulator of tumor cell response to chemotherapy [34]. It has been reported that EGCG enhances the soluble gp130 receptor for IL-6 and suppresses IL-6 signaling in the differentiation of T helper 17 cells [35]. It was also reported that EGCG has a significant anticancer effect on pancreatic cancer, partly by inhibiting the STAT3 signaling pathway [36]. EGCG inhibits the binding of the gp130 receptor to SEA and the phosphorylation of STAT3. Our results suggest that inhibition of the JAK/STAT signaling pathways by the binding of EGCG to SEA may also suppress chronic inflammatory responses. This is the first report to document that EGCG inhibits the binding of the gp130 receptor to SEA and the associated phosphorylation of STAT3.

## 4. Materials and Methods

### 4.1. Materials

SEA (>95% purity; Toxin Technology, Sarasota, FL, USA) was used in all of the studies. SEA stock solution was adjusted to 1 mg/mL with phosphate-buffered saline (PBS) (Gibco, Carlsbad, CA, USA) or distilled water (Gibco), and stored at −20 °C until use. Methylated EGCG [(−)-3″-Me-EGCG and (−)-4″-Me-EGCG] were synthesized according to our previously reported method [37]. Each catechin and methylated EGCG stock solution was adjusted to 300 mM with dimethyl sulfoxide and stored at −20 °C until use. Each stock solution was appropriately diluted in PBS.

### 4.2. Isolation of Mouse Spleen Cells

Russ-10 cell culture medium was prepared by adding 450 mL of RPMI 1640 medium without L-glutamine (Gibco), 50 mL of fetal bovine serum (Gibco), 0.25 mL of 100 mM β-mercaptoethanol (Sigma-Aldrich, St. Louis, MO, USA), 5 mL of 200 mM glutamine (Gibco), 5 mL of antibiotic-antimycotic solution (Gibco), 5 mL of nonessential amino acid mix (Gibco), and 5 mL of sodium pyruvate (Gibco). The spleen was removed from a 6-week-old, female C57BL/6J mouse and washed with ethanol and then cut in the Russ-10 cell culture medium. The cut spleen was homogenized using a BioMasher II (Nippi, Tokyo, Japan). The homogenized spleen was prepared by filtration through a 40-μm cell strainer (BD Labware, San Jose, CA, USA) and centrifuged at 8000× *g*. Red blood cell precipitation was lysed with 10 mL of Ammonium-Chloride-Potassium Lysing Buffer (Gibco) and then centrifuged at 8000× *g*. The supernatant was removed and resuspended in 10 mL of Russ-10 cell culture medium. The viable cells were counted using trypan blue solution and a hemocytometer.

### 4.3. JAK/STAT Signaling Pathway Gene Expression Using Real-Time RT-PCR

The spleen cells were plated in 6-well plates (2.5 × 10^6^ cell/mL) in 4.0 mL of Russ-10 medium. Then, 100 µL of 4.0 mM EGCG (final concentration 0.05 mM) was added, with or without 100 µL of 4 μg/mL SEA (50 ng/mL), followed by incubation at 37 °C in a 5% CO_2_ incubator for 16 h. Total RNA was purified from mouse spleen cells with the RNeasy Mini Kit (Qiagen, Valencia, CA, USA), following the manufacturer’s instructions. Purified RNA was quantified using a K2800 Nucleic Acid Analyzer (Beijing Kaiao Technology Development, Beijing, China). cDNA was synthesized using the PrimeScript RT Reagent Kit (Takara Bio, Shiga, Japan). RT-PCR was performed using the Thermal Cycler Dice^®^ Real Time System (Takara Bio) with TB Green^™^ Premix Ex Taq^™^ II (Takara Bio). The primer pairs of IFN-γ were 5′-CATTGAAAGCCTAGAAAGTCTG-3′ and 5′- CTCATGAATGCATCCTTTTTCG-3′. The primer pairs of STAT3 were 5′-CTACCTCTACCCCGACATTCC-3′ and 5′-GATGAACTTGGTCTTCAGGTACG-3′. The primer pairs of SOCS1 were 5′-GATTCTGCGTGCCGCTCT-3′ and 5′-TGCGTGCTACCATCCTACTC-3′. The primer pairs of hypoxanthine–guanine phosphoribosyl transferase (HPRT) were 5′-GTTGGATACAGGCCAGACTTTGTTG-3′ and 5′- GAGGGTAGGCTGGCCTATAGGCT-3′. Each sample was normalized to HPRT, and relative mRNA levels were determined.

### 4.4. Detection of Phosphorylated STAT3 Using Western Blot Analysis

The spleen cells were placed in 100 mL dishes (1.0 × 10^6^ cell/mL) in 10 mL of Russ-10 medium [20]. Next, 100 µL of 5.0 mM EGCG (final concentration 0.05 mM) was added, with or without 100 µL of 50 μg/mL SEA (500 ng/mL), and then incubated at 37 °C in a 5% CO_2_ incubator for 6 h. Proteins were extracted from mouse spleen cells using the EzRIPA Lysis kit (ATTO, Tokyo, Japan). Protein was quantified using BCA Protein Assay kit (Thermo Fisher Scientific, Waltham, MA, USA). The prepared cell lysates (15 µL) were run on a 10% SDS-PAGE gel and transferred to a polyvinylidene difluoride membrane (ATTO). After blocking the membrane with 0.2% bovine serum albumin solution for 90 min, the membrane was incubated overnight at 4 °C with primary antibodies (1:1000 by volume) to determine the levels of β-actin (Cell Signaling Technology, Danvers, MA, USA), phosphorylated STAT3 Tyr705 (Cell Signaling Technology) and phosphorylated STAT3 Ser727 (Cell Signaling Technology). The membranes were washed and incubated overnight at 4 °C with anti-rabbit immunoglobulin G horseradish peroxidase - conjugated secondary antibody (1:1000 by volume; Cell Signaling Technology). After washing, signals were detected using EzWest LumiOne (ATTO) by a cooled charge-coupled device camera system (Light-Capture II, AE-6981; ATTO). The activated proteins were normalized to β-actin levels. Band intensities were quantified using ImageJ software (National Institutes of Health, Bethesda, MA, USA).

### 4.5. Analysis of Protein and Ligand Interactions by Thermal Shift Assay

SEA and gp130 (Interleukin 6 Signal Transducer, Mouse Recombinant; Creative Biomart, Shirley, NY, USA) was used as the protein, catechin was used as the ligand, and PBS (pH7.2; Gibco) was used as the solvent. The thermal shift assay was performed using a Protein Thermal Shift^™^ Dye Kit (Thermo Fisher Scientific Baltics, Vilnius, Lithuania). SYPR orange dye was diluted into PBS at a ratio of 1:60. Strip tubes (0.1-mL, 8-well) with a clear bottom (Thermo Fisher Scientific) were used with 20 μL of solution per well. SEA (final concentration, 5.0 μg/mL) and ligand (final concentration, 250 μM) were incubated at 37 °C for 2 h. The gp130 solution (final concentration, 1.0 μM) and SYPR orange dye (final concentration, 1:6) were added to the tube and heated from 20 °C to 99 °C with temperature increments of 1 °C/min, using a StepOne Real-Time PCR System (Applied Biosystems, Waltham, MA, USA). The *T*_m_ based on the Boltzmann fitting of the fluorescence/temperature raw data (*T*_m_B values) and the *T*_m_ based on the derivative curve vs. temperature plots (*T*_m_D values) were obtained using Protein Thermal Shift Software v1.0 (Applied Biosystems).

### 4.6. Statistical Analysis

Results were analyzed using the Student’s t-test or one-way analysis of variance, followed by Dunnett’s test, using Microsoft Excel 2016 (Microsoft, Redmond, WA, USA). The significance level was set at *p* < 0.05 and all experiments were replicated at least three times.

## Figures and Tables

**Table 1:** Melting temperature (*T*_m_) of SEA in the presence of catechins.

Sample	*T*_m_ Value (°C)
SEA (control)	62.08 ± 0.07
SEA + EC	61.86 ± 0.05
SEA + EGCG	60.13 ± 0.09 *

Values represent the mean ± SD for three independent experiments. *, *p* < 0.05, compared with control.

**Table 2:** Melting temperature of gp130 in the presence of SEA and EGCG.

Sample	*T*_m_ Value (°C)
gp130 (control)	50.80 ± 0.05
gp130 + SEA	51.96 ± 0.01 *
gp130 + SEA+ EGCG	51.09 ± 0.02 *

Values represent the mean ± SD for three independent experiments. * *p* < 0.05, compared with the control.

## Data Availability

The data presented in this work are available in insert article.
